# Bluetongue, Schmallenberg - what is next? *Culicoides*-borne viral diseases in the 21^st^ Century

**DOI:** 10.1186/1746-6148-10-77

**Published:** 2014-03-31

**Authors:** Constantianus JM Koenraadt, Thomas Balenghien, Simon Carpenter, Els Ducheyne, Armin RW Elbers, Mark Fife, Claire Garros, Adolfo Ibáñez-Justicia, Helge Kampen, Richard JM Kormelink, Bertrand Losson, Wim HM van der Poel, Nick De Regge, Piet A van Rijn, Christopher Sanders, Francis Schaffner, Marianne M Sloet van Oldruitenborgh-Oosterbaan, Willem Takken, Doreen Werner, Frederik Seelig

**Affiliations:** 1 Laboratory of Entomology, Wageningen University, Droevendaalsesteeg 1, 6708 PB Wageningen, The Netherlands; 2 CIRAD, UMR CMAEE, F-34398 Montpellier, France; 3 INRA, UMR1309 CMAEE, F-34398 Montpellier, France; 4 Entomology Group, The Pirbright Institute, Ash Road, Pirbright, Woking GU24 0NF, UK; 5 Avia-GIS, Risschotlei 33, 2980 Zoersel, Belgium; 6 Department of Epidemiology, Crisis organisation and Diagnostics (ECD), Central Veterinary Institute (CVI), part of Wageningen UR, Houtribweg 39, 8221 RA Lelystad, The Netherlands; 7 Genetics and Genomics Group, The Pirbright Institute, Ash Road, Pirbright, Woking, GU24 0NF, UK; 8 Ministry of Economic Affairs, Food and Consumer Safety Authority, Centre for Monitoring of Vectors, Geertjesweg 15, 6706 EA Wageningen, The Netherlands; 9 Federal Research Institute for Animal Health, Südufer 10, 17493 Greifswald - Insel, Riems, Germany; 10 Laboratory of Virology, Wageningen University, Droevendaalsesteeg 1, 6708 PB Wageningen, The Netherlands; 11 Departement des maladies infectieuses et parasitaires, University of Liège, Boulevard de Colonster 20, 4000 Liège, Belgium; 12 Department of Virology, Central Veterinary Institute, Wageningen University and Research Center, Edelhertweg 15, 8219 PH Lelystad, The Netherlands; 13 Enzootic and (re)emerging Diseases, Veterinary and Agrochemical Research Centre, Groeselenberg 99, B-1180 Brussels, Belgium; 14 Department of Virology, Central Veterinary Institute of Wageningen UR (CVI), Houtribweg 39, 8221 RA Lelystad, The Netherlands; 15 The Pirbright Institute, Ash Road, Pirbright, Woking GU24 0NF, UK; 16 National Centre for Vector Entomology, Institute of Parasitology, University of Zürich, Winterthurerstrasse 266a, CH-8057 Zürich, Switzerland; 17 Department of Equine Sciences, Faculty of Veterinary Medicine, Utrecht University, Yalelaan 112, 3584 CM Utrecht, The Netherlands; 18 Institut für Landnutzungssysteme, Medizinische Entomologie, Leibniz-Zentrum für Agrarlandschaftsforchung, Eberswalder Strasse 84, 15374 Müncheberg, Germany

**Keywords:** *Culicoides*, Midge, Schmallenberg virus, Bluetongue virus, Emerging disease, Ecology

## Abstract

In the past decade, two pathogens transmitted by *Culicoides* biting midges (Diptera: Ceratopogonidae), bluetongue virus and Schmallenberg virus, have caused serious economic losses to the European livestock industry, most notably affecting sheep and cattle. These outbreaks of arboviral disease have highlighted large knowledge gaps on the biology and ecology of indigenous *Culicoides* species. With these research gaps in mind, and as a means of assessing what potential disease outbreaks to expect in the future, an international workshop was held in May 2013 at Wageningen University, The Netherlands. It brought together research groups from Belgium, France, Germany, Spain, Switzerland, United Kingdom and The Netherlands, with diverse backgrounds in vector ecology, epidemiology, entomology, virology, animal health, modelling, and genetics. Here, we report on the key findings of this workshop.

## Introduction

In 2011, a novel Orthobunyavirus, provisionally named Schmallenberg virus (SBV), was first detected in cattle blood samples and subsequently in the brains of stillborn, malformed lambs [1]. During the summer of that year, SBV infections caused reductions in milk yield, diarrhoea and fever in adult cattle in Germany and the Netherlands [2]. Later, the involvement of the virus in congenital malformation was demonstrated [3], which remains SBVs primary clinical impact, in addition to evidence of infection in Deer [4] and Camelids [5]. Prior to this incursion, outbreaks of several strains of bluetongue virus (BTV) occurred within the same geographic region [6,7], although it remains uncertain whether the two viruses shared the same route of entry into Europe [8]. Despite both viruses being transmitted by *Culicoides*, clear differences in their pattern of spread have become apparent, as well as differences in their apparent ecology. During a workshop organized in May 2013 at Wageningen University, The Netherlands, these issues were addressed under five different themes: (1) Virus-vector interactions; (2) Sampling methods of *Culicoides*; (3) Diagnostics of *Culicoides* and SBV; (4) Ecology of *Culicoides* and SBV in Europe and (5) Modelling of *Culicoides*-borne disease spread in Europe.

### Virus-vector interactions

The vast majority of detailed studies of *Culicoides*-arbovirus interactions have been conducted using laboratory lines of *C. sonorensis* and *C. nubeculosus* infected with either BTV or the closely related African horse sickness virus (AHSV) [9-11]. Within these restricted models of infection, a series of barriers to dissemination of arboviruses within *Culicoides* have been demonstrated, either directly through immunochemistry, or indirectly from time-series virus infection titres [12,13]. While superficially similar to those barriers defined for mosquitoes, a key apparent difference lies in the lack of salivary gland barriers of infection and release [10,12,14]. This observation has recently been confirmed in *C. sonorensis* infected with SBV [15] and allows evidence of infection in the head of field-collected *Culicoides* to be inferred as being fully disseminated [16,17]. Another relevant characteristic observed in *C. sonorensis* is the ‘leaky gut phenomenon’, which is especially seen when reared at higher temperatures [18]. It results in higher infection rates and indicates that successful dissemination and transmission of a virus is temperature-dependent.

Genetic factors underlying vector competence of *C. sonorensis* for BTV have been explored in the USA and appear to involve a multi-locus determination of susceptibility to fully disseminated infection [19]. These pioneering studies are now being revisited in light of the development of next generation sequencing technology using a systems-based approach of comparative genomics and contemporary genetic mapping strategies. Understanding genetic inheritance of vector competence for BTV is an important driver of the first *de novo* full genome sequence project for the *Culicoides* genus currently being conducted by The Pirbright Institute and the European Bioinformatics Institute (as part of the *Culicoides* Genomics and Transcriptomics Alliance). The production of an accurately annotated genome for *C. sonorensis* will trigger major future advances in comparative genomics in European species, as has been seen in other arthropod vector groups [20]. Understanding the factors driving vector competence of *Culicoides* from the point of view of SBV are likely to be aided by the development of reverse genetics, allowing detailed investigation of infection in the insect host [21].

Following the identification of SBV as a disease-causing agent, several European countries possessing on-going vector surveys were able to identify various *Culicoides* species as potential vectors through the screening of late summer midge pools for the presence of SBV [16,17]. In nine locations in northern Belgium, pools consisting of the heads of *C. obsoletus*, *C. dewulfi* and *C. chiopterus* from August 2011 were found to be positive for SBV [17]. No positive pools were found in the south of Belgium, correlating with a low seroprevalence rate in sheep and cattle at the end of the first vector season. In The Netherlands, individuals of the *Obsoletus* complex (*C. scoticus* and *C. obsoletus*) and *C. chiopterus* were found positive, with estimated field infection rates of 0.56 and 0.14%, respectively. All *C. dewulfi* and *C. punctatus* samples were SBV-negative. The high viral load (as derived from Ct-values in quantitative PCR) and relatively high proportions of infected midges as compared to BTV infection rates could explain the rapid and efficient transmission of SBV in comparison to BTV. Furthermore, the fact that the PCR C_t_ values found in the heads of midges matched closely those obtained from their associated abdomens makes it more certain that SBV had replicated to transmissible levels in these midges, and supports the contention that two species of the Obsoletus Complex, along with *C. chiopterus,* act as natural vectors for SBV [22].

In France, the first cases of SBV were detected in January 2012 and the disease has since spread throughout the country, including to the island of Corsica. Retrospective studies on *Culicoides* from the national surveillance network also implicated *C. chiopterus* as putative vector. In addition, positive pools of *C. dewulfi* and *C. pulicaris* were found (C. Garros, pers. comm.). Interestingly, one positive pool of *C. nubeculosus* was also detected. This species is the only European midge species that has been continuously kept and reared under laboratory conditions [23], but its abundance in France and other northern European countries is relatively low and a recent study of a colony line has demonstrated a low vector competence for SBV [15]. Laboratory studies where explicit estimates of vector competence for SBV can be made for field populations to infer likely influence on dispersal rate are now a priority.

### Sampling methods of *Culicoides*

Adult *Culicoides* are at present most commonly collected in the field using Onderstepoort Veterinary Institute (OVI) light-suction traps that utilise a UV light source to attract Insects. A key criticism of their use is that it remains unclear why *Culicoides* are attracted to light and catches have been repeatedly demonstrated to be unrepresentative of collections made on hosts at the same site [24]. In addition to light-suction traps, unbaited suction traps are also used such as the 11 metre suction trap network maintained as a national capability by the Rothamsted Insect Survey [25]. Studies from Belgium revealed that, during the same year, two Rothamsted suction traps collected 21 and 25 different *Culicoides* species, respectively. One OVI trap and 88 emergence traps located in the first site with a Rothamsted suction trap collected 17 and 12 species, respectively. Using sheep as bait for trapping, approximately 50% of the midges captured at a site in The Netherlands were *C. chiopterus* and around 40% were *C. obsoletus*[26]. Interestingly, *C. obsoletus* was by far the most common species found around horses. The average successful *Culicoides* feeding rate was 35–40%. *Culicoides* activity was greatest around sunset, less at sunrise and they were only rarely trapped in the afternoon or at night on hosts [27].

Adult sampling can also be conducted using emergence trapping from sites of larval development. In studies from Germany, 22 *Culicoides* species were collected from emergence traps, including species of the Obsoletus complex occurring in Germany (plus *C. dewulfi*) and four species of the *Culicoides* subgenus Pulicaris group (*C. pulicaris*, *C. punctatus*, *C. newsteadi*, *C. deltus*). Depending on the substrate onto which the emergence trap was placed, abundance per 48 hours of trapping ranged from very few to up to 2,000 individuals. These results demonstrated that the most important habitat types for *Culicoides* are bog land (primarily for the Obsoletus complex), wet forest areas (especially for Pulicaris complex) and animal faeces. It was noted that emergence traps on dung caught a greatest abundance of *Culicoides* in March and April, when emergence commonly occurs from these overwintering habitats. Dung containing hay (from dung heaps) was found to be highly productive as a *Culicoides* larval habitat and also more important than loose animal droppings.

The OVI light-suction trap remains a standard tool for *Culicoides* detection that has a significant role in the detection of vector-low periods allowing animal movement restrictions to be reduced. However, as *Culicoides* ultimately find their hosts based on odours, it was concluded that more research effort should be put into the development and addition of semiochemicals as lures to trapping systems. Ideally, such trapping systems would be sensitive in low *Culicoides* density situations, thus making them more suitable for surveillance during winter periods.

### Diagnostics of *Culicoides* and SBV

Accurate morphological identification of *Culicoides* is considered challenging, requiring significant expertise and is restricted to relatively few entomologists in Europe. Online resources for identification (e.g. http://www.culicoides.net; http://avabase.cirad.fr/) and taxonomic training [28] may reduce this problem. Some morphologically similar species of *Culicoides* can also now be accurately identified to species level based on DNA marker regions such as the CO-I and ITS-2 [29,30]. High-throughput systems for this have been developed that are being applied to large-scale surveillance sampling in France [31]. As an alternative to these molecular tools, matrix-assisted laser desorption/ionization-time of flight mass spectrometry (MALDI-TOF MS) has gained interest for high throughput identification of microorganisms and has been established to identify adults and larvae of *Culicoides*[32,33]. This protein-profiling approach revealed an accuracy of 98.9% when using 1,200 randomly selected *Culicoides* specimens. Whether this technology will become available at large scale for surveillance programs remains to be seen, especially in the light of the rapidly decreasing costs of DNA sequencing.

Sequence comparisons of SBV isolates from different hosts have revealed high variability in the M-region of the virus genome. This was especially apparent between an ovine brain isolate and other lamb brain isolates and cattle blood isolates. This variability may be due to adaptation to host or vector tissues and indicates variation within the epidemic. The origins of atypical variants remain to be investigated. Reverse genetics approaches have been and are being developed to identify cellular pathways involved in vector competence with selected virus mutants. Besides these molecular approaches, classic transmission experiments, where potential hosts are exposed to infected insects and naive blood-feeding insects are exposed to infected hosts remain essential for assessing the role of vectors in the natural transmission cycle.

### Ecology of *Culicoides* and SBV in Europe

The capability of *Culicoides* species to overwinter and serve as reservoir for new infections during the next year is of relevance to the transmission of both BTV and SBV. Until quantitative evidence is provided otherwise, possible overwintering strategies in the vector, host or via alternative transmission pathways should all be considered. The detection of SBV RNA in field caught nulliparous *Culicoides* in Poland [34] is an important epidemiological finding and resembles a previous study that detected BTV in *C. sonorensis* larvae in the USA [35]. This implies that transovarial transmission could represent an alternative means of overwintering for SBV. Confirmation and isolation of live virus from adults is required, however, given the repeated failure of previous studies to replicate this phenomenon in *C. sonorensis* under laboratory conditions with BTV [10,36].

In The Netherlands, Denmark and Switzerland, first trials were performed with honey-baited FTA cards that were deployed in the vicinity of *Culicoides* traps. The goal of this was to detect the circulation of SBV at an early time of the year in a given area. Based on the expectoration of virus in the saliva onto the FTA preservation cards, trials using this technique in Australia have been able to successfully recover arboviruses from collected mosquitoes [37]. At present, this approach is being refined for virus surveillance in *Culicoides* although initial studies in the laboratory appear to show that these are unlikely to be effective [15]*.*

Host preference of *Culicoides* is of significant importance in determining the probability of arbovirus transmission. Recent studies from France using sticky traps on animals have shown a clear preference of *Culicoides* midges (*C. obsoletus s.s.*, *C. dewulfi* and *C. scoticus*) to bite horses over sheep, chicken, goats or cattle, even after correcting for body surface or weight) [38]. *Culicoides* midges could be taken from a layer of petroleum jelly on panels applied to livestock and were still useful for molecular biology purposes [30]. Catch rates from an OVI trap used in parallel to the sticky traps on animals over-estimated the biting rate of *C. obsoletus* and under-estimated the biting rate of *C. dewulfi* on a horse. This supports earlier findings demonstrating that OVI traps are not representative of actual feeding events on a host [24]. Although most biting midges were captured outdoors, *C. obsoletus* showed some degree of endophagy. An overall increase of endophagy was observed in autumn. Again, the use of semio-chemicals in traps was recommended for surveillance purposes. Despite the limitations of the current trap types, data from traps remain useful for modelling purposes.

### Modelling of *Culicoides*-borne disease spread in Europe

Predicting the spread of *Culicoides*-borne diseases is of great importance for preventing and reducing disease burdens. In the case of BTV, a trend-surface analysis coupled to a simultaneous autoregressive model has been used to assess the wave-front velocity at which BTV spread in France [39,40]. In the context of restricted animal movement, the wave-front velocities of BTV-1 and BTV-8 were similar: 5.4 and 5.6 km per day, respectively. Ecological factors associated with vector abundance and activity (meteorologically related variables and elevation), as well as with host availability, were the most important drivers of this spread. The spread of SBV was probably faster than of BTV, but in this case there were no animal movement restrictions.

Other models, such as atmospheric dispersion models, have been employed to simulate vector movement and the risk of wind-borne vector introduction. These are highly effective at predicting incursions of infected *Culicoides* across water bodies and have been used to both predict movement and also in retrospective analyses of spread [41]. Modelling movement of *Culicoides* over land has been more challenging due to the difficulties of both predicting behaviour at a farm level and the greater complexity of air turbulence over landscape. Currently studies are focussing on the use of mark recapture techniques to define *Culicoides* dispersal at a local scale [42]. These studies are known, however, to invariably violate a rule of dispersal studies in significantly influencing the behaviour of released insects due to inflicting physical damage. An alternative approach may lie in the use of genetic or genomic markers to track population movement.

A third approach of modelling BTV spread is currently using Random Regression Forest modelling. ‘Random forests’ is an ensemble of decision trees using 74 variables (such as land cover, human population density and water capacity). Modelling efforts thus far have shown that the probability of vector occurrence is a good predictor of abundance at the used spatial resolution of 5 × 5 km. Abundance of *C. imicola* is mostly driven by rain, whereas *C. obsoletus* is more affected by temperature. The proposed methodology can be used as an input to TIR models and R_0_ models and will become available to the general public as part of the VECMAP^TM^ software.

## Conclusions

There was a general consensus within the group that future studies should move beyond simple monitoring of adult *Culicoides* populations and address fundamental aspects of vector competence in particular, as it has been shown that *Culicoides* species and populations contribute differentially to disease epidemiology. With respect to surveillance, a key question was to what degree a sampling strategy should reflect activity of *Culicoides* on a host. While a non-representative, but very sensitive surveillance method (e.g. the use of OVI light-suction traps) might be suitable for defining vector-free periods (as defined by EU legislation), the degree to which these data could be used as a proxy for biting rate in modelling of transmission was not well understood. The proliferation of trap types used for surveillance in Europe was seen as presenting difficulties in maintaining a coherent understanding of *Culicoides* abundance and distribution, as in other vector groups. Besides these limitations in the surveillance of adult *Culicoides* populations, the group concluded that data on the larval biology and ecology of midge species, e.g. on their preferred breeding habitats and substrates, is sparse and limits our options for efficient and targeted vector management.

To answer the question posed in the title, it was concluded that the emergence of novel viruses cannot be predicted except in the very broadest sense. Assessments of potential outbreak risks in Europe for other known *Culicoides*-borne viruses, such as African horse sickness virus, are still in their infancy [43]. A key current question is whether SBV and the BTV-8 strain shared the same route of entry into northern Europe and if so whether this route remains open [8]. The incursions of Schmallenberg and bluetongue have led to studies that have generated fundamental insights into the ecology of *Culicoides*-borne diseases. In order to retain this capacity under the budget constraints likely to be imposed on research in Europe, transnational funding was seen as being highly appropriate. A model in this respect is the current EDENnext project (http://www.edenext.eu/), which has brought together a vast range of expertise from 48 institutes across Europe to understand vector-borne disease epidemiology.

## Competing interests

The authors declare that they have no competing interests.

## Authors’ contributions

CJMK, FS and WT organized the workshop. CJMK and FS prepared the first synthesized draft of the manuscript. All other authors provided input to the manuscript in the form of brief written summaries of their oral contributions to the workshop.

## References

[B1] Hoffmann B Scheuch M Hoeper D Jungblut R Holsteg M Schirrmeier H Eschbaumer M Goller KV Wernike K Fischer M Mettenleiter TC Beer M Novel Orthobunyavirus in Cattle, Europe, 2011 Emerg Infect Dis 2012 18 3 469 472 10.3201/eid1803.111905 22376991 PMC3309600

[B2] van den Brom R Luttikholt SJM Lievaart-Peterson K Peperkamp NHMT Mars MH van der Poel WHM Vellema P Epizootic of ovine congenital malformations associated with Schmallenberg virus infection Tijdschr Diergeneeskd 2012 137 2 106 111 22393844

[B3] Garigliany MM Hoffmann B Dive M Sartelet A Bayrou C Cassart D Beer M Desmecht D Schmallenberg virus in calf born at term with porencephaly, Belgium Emerg Infect Dis 2012 18 6 1005 1006 22607989 10.3201/eid1806.120104 PMC3358169

[B4] Barlow A Green P Banham T Healy N Serological confirmation of SBV infection in wild British deer Vet Rec 2013 172 16 429 429 10.1136/vr.f2438 23603727

[B5] Jack C Anstaett O Adams J Noad R Brownlie J Mertens P Evidence of seroconversion to SBV in camelids Vet Rec 2012 170 23 603 603 22696474 10.1136/vr.e3939

[B6] Carpenter S Wilson A Mellor PS Culicoides and the emergence of bluetongue virus in northern Europe Trends Microbiol 2009 17 4 172 178 10.1016/j.tim.2009.01.001 19299131

[B7] Takken W Verhulst N Scholte EJ Jacobs F Jongema Y van Lammeren R The phenology and population dynamics of Culicoides spp. in different ecosystems in The Netherlands Prev Vet Med 2008 87 1-2 41 54 10.1016/j.prevetmed.2008.06.015 18639947

[B8] Carpenter S Groschup MH Garros C Felippe-Bauer ML Purse BV *Culicoides* biting midges, arboviruses and public health in Europe Antivir Res 2013 100 102 113 10.1016/j.antiviral.2013.07.020 23933421

[B9] Mellor PS *Culicoides*: vectors, climate change and disease risk Vet Bull 1996 66 4 301 306

[B10] Mellor PS Carpenter S White DM Mellor PS, Baylis M, Mertens P Bluetongue virus in the insect host Biology of Animal Infections: Bluetongue 2009 Oxford, UK: Elsevier, Academic Press 295 320

[B11] Tabachnick WJ *Culicoides variipennis* and bluetongue-virus epidemiology in the United States Annu Rev Entomol 1996 41 23 43 10.1146/annurev.en.41.010196.000323 8546447

[B12] Fu H Leake CJ Mertens PPC Mellor PS The barriers to bluetongue virus infection, dissemination and transmission in the vector, Culicoides variipennis (Diptera: Ceratopogonidae) Arch Virol 1999 144 4 747 761 10.1007/s007050050540 10365165

[B13] Sieburth PJ Nunamaker CE Ellis J Nunamaker RA Infection of the midgut of *Culicoides variipennis* (Diptera, Ceratopogonidae) with bluetongue virus J Med Entomol 1991 28 1 74 85 1851848 10.1093/jmedent/28.1.74

[B14] Bowne JG Jones RH Observations on bluetongue virus in salivary glands of an insect vector *Culicoides variipennis* Virology 1966 30 1 127 10.1016/S0042-6822(66)81016-4 5911888

[B15] Veronesi E Henstock M Gubbins S Batten C Manley R Barber J Hoffmann B Beer M Attoui H Mertens PPC Carpenter S Implicating *Culicoides* biting midges as vectors of Schmallenberg virus using semi-quantitative RT-PCR PLoS One 2013 8 3 e57747 10.1371/journal.pone.0057747 23520481 PMC3592918

[B16] Elbers ARW Meiswinkel R van Weezep E van Oldruitenborgh-Oosterbaan MMS Kooi EA Schmallenberg virus in Culicoides spp. biting midges, the Netherlands, 2011 Emerg Infect Dis 2013 19 1 106 109 10.3201/eid1901.121054 23260040 PMC3558002

[B17] De Regge N Deblauwe I Deken R Vantieghem P Madder M Geysen D Smeets F Losson B van den Berg T Cay AB Detection of Schmallenberg virus in different Culicoides spp. by real-time RT-PCR Transbound Emerg Dis 2012 59 6 471 475 10.1111/tbed.12000 23025501

[B18] Mellor PS Rawlings P Baylis M Wellby MP Effect of temperature on African horse sickness virus infection in Culicoides Arch Virol 1998 14 155 163 10.1007/978-3-7091-6823-3_15 9785504

[B19] Tabachnick WJ Genetic control of oral-susceptibility to infection of *Culicoides variipennis* with bluetongue virus Am J Trop Med Hyg 1991 45 6 666 671 1662471 10.4269/ajtmh.1991.45.666

[B20] Severson DW Behura SK Mosquito genomics: progress and challenges Annu Rev Entomol 2012 57 143 166 10.1146/annurev-ento-120710-100651 21942845

[B21] Elliott RM Blakqori G van Knippenberg IC Koudriakova E Li P McLees A Shi XH Szemiel AM Establishment of a reverse genetics system for Schmallenberg virus, a newly emerged orthobunyavirus in Europe J Gen Virol 2013 94 851 859 10.1099/vir.0.049981-0 23255627 PMC3709688

[B22] Elbers ARW Meiswinkel R Van Weezep E Sloet vanOldruitenborgh-Oosterbaan MM Kooi EA Schmallenberg virus detected in *Culicoides* spp. biting midges, the Netherlands, 2011 Emerg Infect Dis 2013 19 106 109 10.3201/eid1901.121054 23260040 PMC3558002

[B23] Boorman J Maintenance of laboratory colonies of *Culicoides variipennis* (Coq), *Culicoides nubeculosus* (Mg) and *Culicoides riethi* Kieff. (Diptera: Ceratopogionidae) Bull Entomol Res 1974 64 371 377 10.1017/S0007485300031254

[B24] Carpenter S Szmaragd C Barber J Labuschagne K Gubbins S Mellor P An assessment of Culicoides surveillance techniques in northern Europe: have we underestimated a potential bluetongue virus vector? J Appl Ecol 2008 45 4 1237 1245

[B25] Sanders CJ Shortall CR Gubbins S Burgin L Gloster J Harrington R Reynolds DR Mellor PS Carpenter S Influence of season and meteorological parameters on flight activity of Culicoides biting midges J Appl Ecol 2011 48 6 1355 1364 10.1111/j.1365-2664.2011.02051.x

[B26] Griffioen K van Gemst DBJ Pieterse MC Jacobs F van Oldruitenborgh-Oosterbaan MMS *Culicoides* species associated with sheep in The Netherlands and the effect of a permethrin insecticide Vet J 2011 190 2 230 235 10.1016/j.tvjl.2010.10.016 21169040

[B27] van der Rijt R van den Boom R Jongema Y van Oldruitenborgh-Oosterbaan MMS *Culicoides* species attracted to horses with and without insect hypersensitivity Vet J 2008 178 1 91 97 10.1016/j.tvjl.2007.07.005 17728164

[B28] Mathieu B Cetre-Sossah C Garros C Chavernac D Balenghien T Carpenter S Setier-Rio M-L Vignes-Lebbe R Ung V Candolfi E Delecolle JC Development and validation of IIKC: an interactive identification key for Culicoides (Diptera: Ceratopogonidae) females from the Western Palaearctic region Parasites & Vectors 2012 5 137 10.1186/1756-3305-5-137 22776566 PMC3483010

[B29] Cetre-Sossah C Baldet T Delecolle JC Mathieu B Perrin A Grillet C Albina E Molecular detection of *Culicoides* spp. and *Culicoides imicola*, the principal vector of bluetongue (BT) and African horse sickness (AHS) in Africa and Europe Vet Res 2004 35 3 325 337 10.1051/vetres:2004015 15210081

[B30] Nolan DV Carpenter S Barber J Mellor PS Dallas JF Mordue AJ Piertney SB Rapid diagnostic PCR assays for members of the *Culicoides obsoletus* and *Culicoides pulicaris* species complexes, implicated vectors of bluetongue virus in Europe Vet Microbiol 2007 124 1–2 82 94 17478060 10.1016/j.vetmic.2007.03.019

[B31] Mathieu B Delecolle J-C Garros C Balenghien T Setier-Rio M-L Candolfi E Cetre-Sossah C Simultaneous quantification of the relative abundance of species complex members: Application to *Culicoides obsoletus* and *Culicoides scoticus* (Diptera: Ceratopogonidae), potential vectors of bluetongue virus Vet Parasitol 2011 182 2–4 297 306 21715095 10.1016/j.vetpar.2011.05.052

[B32] Kaufmann C Schaffner F Ziegler D Pfluger V Mathis A Identification of field-caught *Culicoides* biting midges using matrix-assisted laser desorption/ionization time of flight mass spectrometry Parasitology 2012 139 2 248 258 10.1017/S0031182011001764 22008297

[B33] Kaufmann C Ziegler D Schaffner F Carpenter S Pflueger V Mathis A Evaluation of matrix-assisted laser desorption/ionization time of flight mass spectrometry for characterization of *Culicoides nubeculosus* biting midges Med Vet Entomol 2011 25 1 32 38 10.1111/j.1365-2915.2010.00927.x 21118284

[B34] Larska M Lechowski L Grochowska M Zmudzinski JF Detection of the Schmallenberg virus in nulliparous Culicoides obsoletus/scoticus complex and C. punctatus-The possibility of transovarial virus transmission in the midge population and of a new vector Vet Microbiol 2013 166 3–4 467 473 23928121 10.1016/j.vetmic.2013.07.015

[B35] White DM Wilson WC Blair CD Beaty BJ Studies on overwintering of bluetongue viruses in Insects J Gen Virol 2005 86 453 462 10.1099/vir.0.80290-0 15659765

[B36] Nunamaker RA Sieburth PJ Dean VC Wigington JG Nunamaker CE Mecham JO Absence of transovarial transmission of bluetongue virus in *Culicoides variipennis* - immunogold labeling of bluetongue virus antigen in developing oocytes from *Culicoides variipennis* (Coquillet) Comp Biochem Physiol A Physiol 1990 96 1 19 31 10.1016/0300-9629(90)90036-R 1975536

[B37] Hall-Mendelin S Ritchie SA Johansen CA Zborowski P Cortis G Dandridge S Hall RA van den Hurk AF Exploiting mosquito sugar feeding to detect mosquito-borne pathogens Proc Natl Acad Sci U S A 2010 107 25 11255 11259 10.1073/pnas.1002040107 20534559 PMC2895145

[B38] Viennet E Garros C Lancelot R Allene X Gardes L Rakotoarivony I Crochet D Delecolle JC Moulia C Baldet T Balenghien T Assessment of vector/host contact: comparison of animal-baited traps and UV-light/suction trap for collecting Culicoides biting midges (Diptera: Ceratopogonidae), vectors of Orbiviruses Parasite Vector 2011 4 119 10.1186/1756-3305-4-119 PMC3145584 21707980

[B39] Pioz M Guis H Calavas D Durand B Abrial D Ducrot C Estimating front-wave velocity of infectious diseases: a simple, efficient method applied to bluetongue Vet Res 2011 42 60 10.1186/1297-9716-42-60 21507221 PMC3090993

[B40] Pioz M Guis H Crespin L Gay E Calavas D Durand B Abrial D Ducrot C Why did bluetongue spread the way it did? Environmental factors influencing the velocity of bluetongue virus serotype 8 epizootic wave in France Plos One 2012 7 8 e43360 10.1371/journal.pone.0043360 22916249 PMC3419712

[B41] Burgin LE Gloster J Sanders C Mellor PS Gubbins S Carpenter S Investigating incursions of bluetongue virus using a model of long-distance *Culicoides* biting midge dispersal Transbound Emerg Dis 2013 60 3 263 272 10.1111/j.1865-1682.2012.01345.x 22672434

[B42] Kirkeby C Bodker R Stockmarr A Lind P Heegaard PMH Quantifying dispersal of European *Culicoides* (Diptera: Ceratopogonidae) vectors between farms using a novel Mark-Release-Recapture technique Plos One 2013 8 4 7 10.1371/journal.pone.0061269 PMC3632603 23630582

[B43] de Vos CJ Hoek CA Nodelijk G Risk of introducing African horse sickness virus into the Netherlands by international equine movements Prev Vet Med 2012 106 2 108 122 10.1016/j.prevetmed.2012.01.019 22341773

